# Bilateral rectus muscle turning-over for complicated and eventrated abdominal wall hernias: results of a novel method

**DOI:** 10.1590/acb393624

**Published:** 2024-08-16

**Authors:** Gábor Martis, Renáta Laczik, Norbert Németh, Gabriella Martis, László Damjanovich

**Affiliations:** 1 University of Debrecen – Faculty of Medicine – Department of Surgery – Debrecen – Hungary.; 2 University of Debrecen – Faculty of Medicine – Department of Angiology – Debrecen – Hungary.; 3 University of Debrecen – Faculty of Medicine – Department of Operative Techniques and Surgical Research – Debrecen – Hungary.; 4 University of Debrecen – Faculty of Medicine – Medical School – Debrecen – Hungary.

**Keywords:** Rectus Abdominis, Hernia, Abdominal, Abdominal Wound Closure Techniques

## Abstract

**Purpose:**

We present a technique for covering large midline loss of abdominal wall using a novel method by autologous tissues.

**Methods:**

Twenty-two patients (body mass index = 35,6 ± 6,9 kg/m^2^) were involved in the prospective cohort study. Acute and elective cases were included. The gap area was 450.1 ± 54 cm^2^. The average width of the midline gap was 16,3 ± 3,2 cm. The rectus muscles were mobilized from its posterior sheath. Both muscles were turned by180º medially, so that the complete abdominal wall gap could be covered without considerable tension. Changes in intra-abdominal pressure, quality of life and hernia recurrency were determined.

**Results:**

There was no significant increase in the intra-abdominal pressure. Wound infection and seroma occurred in four cases. Bleeding occurred in one case. Pre- and post-operative quality of life index significantly improved (23 ± 13 vs. 47 ± 6; p = 0,0013). One recurrent hernia was registered. The procedure could be performed safely and yielded excellent results. The method was applied in acute cases. The intact anatomical structure of rectus muscles was essential.

**Conclusions:**

The midline reconstruction with bilateral turned-over rectus muscles provided low tension abdominal wall status, and it did not require synthetic mesh implantation.

## Introduction

**Figure 1:**
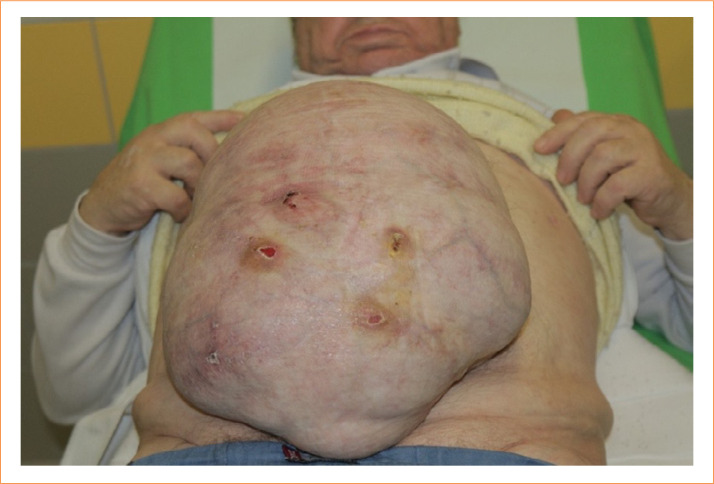
Eventrated, loss of abdominal wall domain, one-year after series (11 operations in three months) of intra-abdominal and retroperitoneal surgeries. Prior to the abdominal wall reconstruction, the patient (65) was operated on necrotizing pancreatitis and extended retroperitoneal necrosis. The maximum horizontal diameter of the hernia was 19 cm. The abdominal wall hernia area was 576 cm^2^. The medial edges of rectus abdominis muscles were in the medioclavicular line. These patients’ main complaint, apart from the aesthetic dissatisfaction, was the load incapacity of the abdominal wall. Low tension reconstruction of this debilitating status is an explicit challenge for surgeons.

Source: Elaborated by the authors.

The disintegrated midline of the abdominal wall usually heals by the second intention between the medial margins of the rectus abdominis muscles progressively lateralizing from each other after open abdomen and intra-abdominal/retroperitoneal series of operations^1^^–^ 4. The progressive lateralization of the rectus abdominis muscles leads to midline loss of abdominal wall domain and eventration. This condition renders patients disabled not only aesthetically, but also in terms of functionality (Fig. 1). In such cases, different types of biological or synthetic meshes can be applied with a wide overlap, in order to complete and/or reinforce the abdominal wall^5^^–^ 9.

We present a prospective cohort study conducted for acute and elective reconstruction of eventrated, loss of domain hernia developing after a series of retroperitoneal surgeries, or open abdomen treatments. The essence of this method is to *recreate* the missing *medial* anatomical structures without tension by *releasing* both *rectus abdominis* muscles from the posterior fascia of the rectus sheath and to *turning the muscles* in 180º towards the midline. This technique uses only the preserved hernia sac and the bilateral rectus muscles for the reconstruction.

The primary aim of this publication was to present the applied surgical technique. The secondary aim was to present early and late results and complications of the intervention, as well as hernia recurrence during the follow-up period. The authors assumed if they can mobilize the rectus abdominis muscles properly, the extended midline gap can be covered without tension, without implanting a synthetic or biological mesh. The excellent blood supply and anatomical position of the rectus abdominis muscles have already been exploited in several surgical procedures^10^^,^ 11.

## Methods

### Study planning

This study was planned as a consecutive prospective cohort study and carried out at the University of Debrecen, School of Medicine, Department of Surgery, between January 1, 2016 and December 31, 2020. A total of 31 patients developed abdominal wall hernia due to open abdomen or series of intra-abdominal surgeries during enrollment, however only 22 of them met eligibility criteria. The follow-up time was 25 ± 9 (3–48) months.

The procedures were conducted in accordance with the ethical standards of the Ethical Committee of the authors’ institution, permission number DE RKEB/IKEB: 4,599, and the national ethical-professional permission number is ETT TUKEB 51036-2/EKU. The method is in accordance with the ethical standards of the Helsinki Declaration.

### Inclusion and exclusion criteria

The most important inclusion criterion was the intact structure of the bilateral rectus abdominis and the lateral abdominal wall, confirmed or excluded by abdominal wall computed tomography assays. Inclusion and exclusion criteria for the study are listed in Table 1.

**Table 1:** Inclusion and exclusion criteria.

	Inclusion		Exclusion
1	Obtained and signed informed consent	1	NYHA III or IV stage
2	Age ≥ 18 years old	2	ASA IV stage
3	Eventerated, loss of domain midline hernia	3	Concurrent malignancy
4	Series of abdominal operations	4	Concurrent chemo- and/or irradiation therapy
5	Open abdomen and/or VAC treatment	5	Decompensated hepatic cirrhosis
6	Last abdominal surgery ≥ one year	6	Therapy resistant ascites
7	CT evaluation of the abdominal wall	7	Acute pancreatitis
8	Intact rectus muscle and lateral domain	8	Chronic pancreatitis with acute exacerbations
9	At least 10 cm of width of abdominal gap	9	Fever of unknown origin
10	Full length of midline gap	10	Pneumonia
11	No signs of original abdominal disorder(s)	11	Chronic alcoholism
12	No sign of retroperitoneal abscess	12	Gravidity or lactation
13	No sign of entero-cutaneous fistula	13	Current drug addiction and dependency
14	CDCP I or II abdominal wall environment		
15	CDCP III, IV, peritonitis, bowel perforation		
16	Mechanical bowel obstruction		

VAC: vacuum assisted closure; CT: computer tomography; CDCP: Centers of Disease Control and Prevention; NYHA: New York Heart Association; ASA: American Society of Anesthesiologists. Source: Elaborated by the authors.

Precise length, width of the midline gap, and the width and thickness of both rectus abdominis muscles, as well as body mass index values, were measured (Table 2).

**Table 2:** Acute and elective patients’ clinical data.

Patients’ clinical data	Acute (6)	Elective (16)	All (22)
Average age (y)	53.3 ± 7.1	41.3 ± 5.3	44.5 ± 12.57
Gender (m/f)	3m/3f	13m/3f	16m/3f
Diabetes mellitus (n)	3	7	10 (45.4%)
Chronic obstructive pulmonary disease (n)	0	3	3 (13.6%)
Active smoking (n)	2	10	12 (54.4%)
Hypertension (n)	1	2	3 (13.6٪)
Coronary heart disease	0	2	2 (9.1٪)
Average length of the defect (cm)	29.9 ± 4.2	28.9 ± 4.3	29 ± 2.46
Average width of the defect (cm)	16.3 ± 3.2	14.1 ± 2.4	14.75 ± 2.22
Average abdominal wall area (cm^2^)	439 ± 50.2	489.3 ± 47.2	450.1 ± 54.3
Average body mass index (kg/m^2^) prior to reconstruction	35.3 ± 2.5	31.7 ± 4.1	33.3 ± 5.1
Average body mass index (kg/m^2^) one-year after the reconstruction	35.9 ± 4.1	32.9 ± 3.3	35.4 ± 3.2

Source: Elaborated by the authors.

### Surgical method

Surgeries were performed under general anesthesia and in total muscle relaxation. After total midline, *or* lower “bay-leaf” transverse (between the iliac crests) incision and usual preparation of the hernia sac, the greatest longitudinal and horizontal diameters of the abdominal wall defect, as well as its perimeter, were measured.

**Figure 2:**
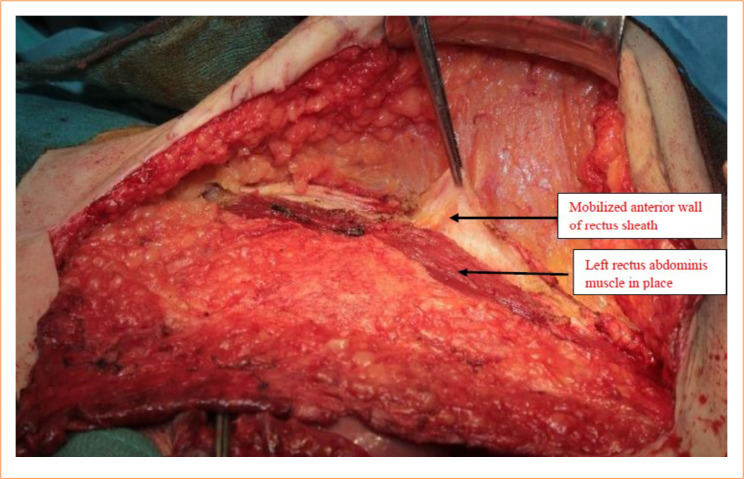
Incision of the lateral margin of the left rectus sheath. The forceps hold the margin of the incised rectus sheath. The lateral margin of the rectus muscle is well visible. Notice that the incision goes from muscle origin to its insertion.

Source: Elaborated by the authors.

**Figure 3:**
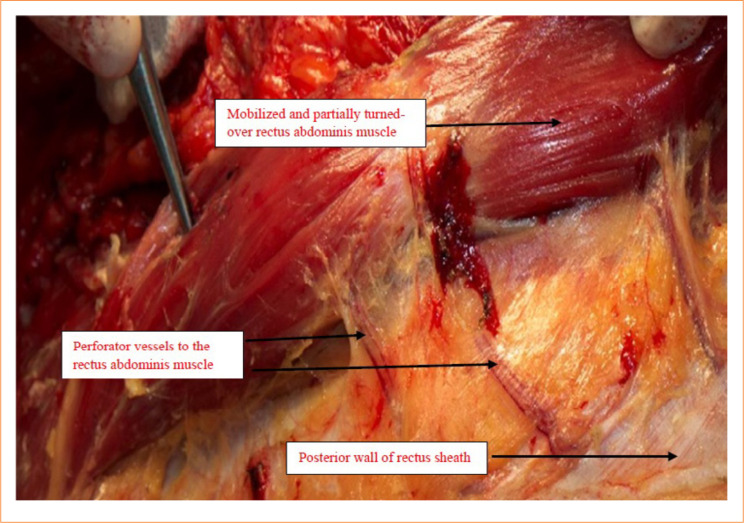
The left rectus muscle is being mobilized from the dorsal rectus sheath and turned over towards the midline, like opening a book. The incision of the lateral margin of the rectus sheath is well visible laterally. Notice the appropriate color of the muscle. The perforator segmental vessels are before dissection and ligation.

Source: Elaborated by the authors.

The next step was the identification of the lateral margin of the bilateral rectus abdominis muscles. After that, the anterior fascia of the rectus muscles were incised from origin to insertion, thus revealing the rectus abdominis muscle (Fig. 2). Starting from the lateral margin, the muscle was detached from its dorsal rectus sheath fascia, except for a 2-cm medial margin. All segmental perforating vessels were ligated during mobilization. Segmental nerves coming from the 7–12 thoracal spinal nerves to the rectus abdominis muscle were partially dissected together with the perforator vessels. The upper (7 and/or 8) and lower (11 and/or 12) nerves remained intact depending on the extension of rectus release. The origin of the muscle was detached from the 7^th^ and 8^th^ costal cartilage. The medial side of the muscle origin (xiphoid process, 5^th^, and 6^th^ costal cartilage) remained intact. The superior and inferior epigastric arteries were carefully spared. The lateral side of the muscle insertion was also released from the symphysis. The released muscles were turned over by 180º medially (Fig. 3).

**Figure 4:**
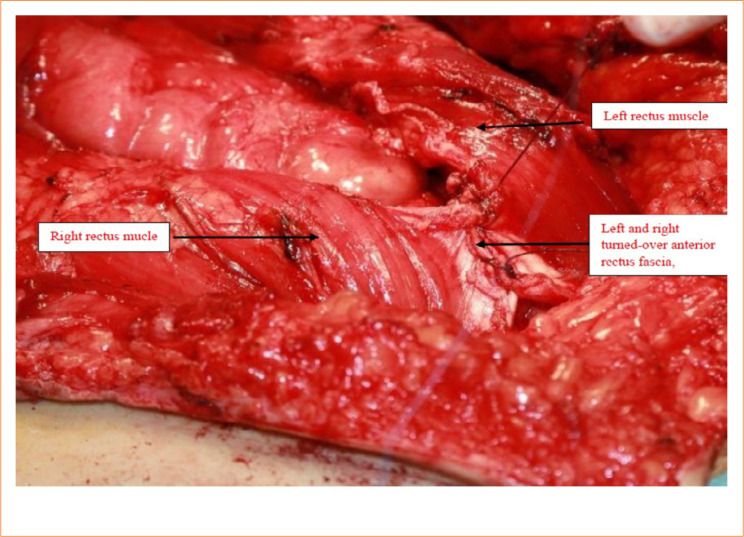
Recreation of the midline. By turning over the rectus muscle, the anterior wall of the original rectus sheath forms the dorsal wall fascia. The rectus muscles turned over on both sides is well seen.

Source: Elaborated by the authors.

**Figure 5:**
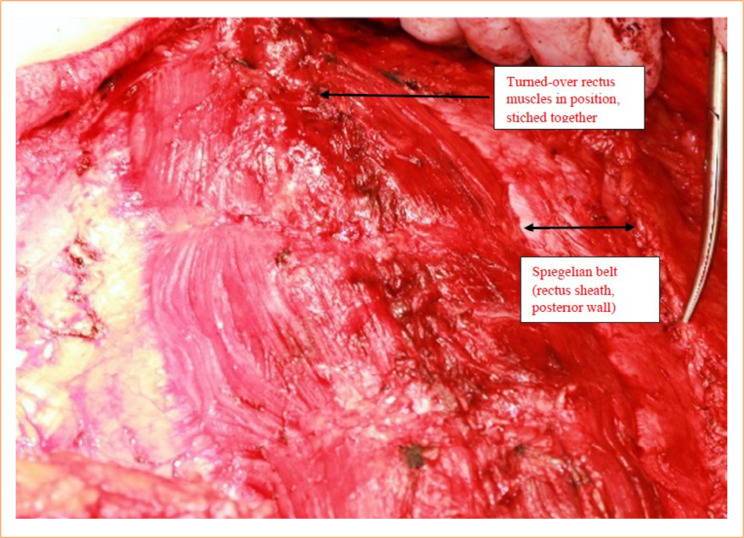
Reconstruction is completed. Note the viable, not strained rectus muscles sutured in the middle. The end of the scissor’s points at the semicircular line, there is a sutured down to the posterior fascia of the rectus sheath (Spiegelian belt). An area of 5-cm width between the semicircular line and the lateral edge of the rectus muscle is well visible. The area is intact, no signs of any damage or considerable laxity, so it does not require reinforcement.

Source: Elaborated by the authors.

The abdominal wall defect was reconstructed as follows: the spared hernia sac was cut for the suitable size and closed in the midline with 3/0 stitches. From the symphysis to the xiphoid process, the originally anterior (now in posterior position) rectus sheath fascia was closed by 3/0 stitches (Fig. 4). The rectus muscles turned-over beside each other were sutured in the midline using 3/0 interrupted absorbable stitches. Laterally, the external and internal oblique abdominal muscle fascia (semicircular line) were stitched with 3/0 absorbable sutures in a running fashion to the dorsal fascia of the rectus sheath (Fig. 5).

**Figure 6:**
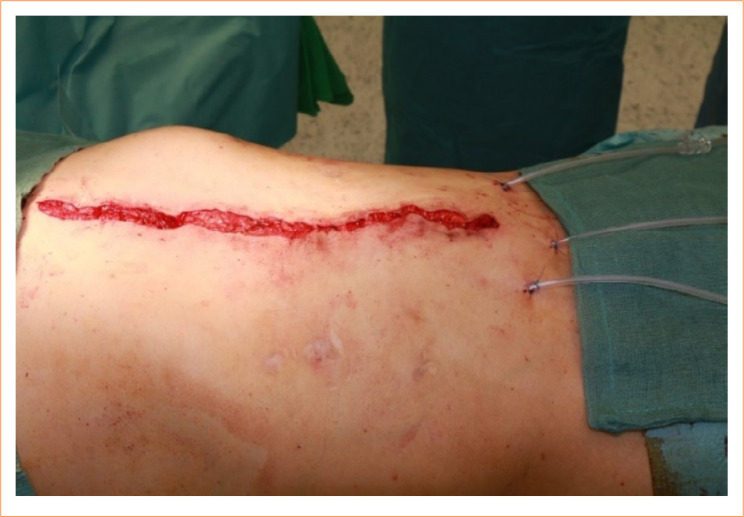
The reconstructed abdominal wall. The necessary amount of superfluous skin and subcutis is removed. Three drains are left in place for proper drainage.

Source: elaborated by the authors.

The surgery site was rinsed with 2:1 solution of H_2_O_2_ and povidone iodine. Three suction drains were left at the operating site (Fig. 6). The superfluous skin and subcutis were excised aesthetically. Before skin closure, the subcutaneous layer was anchored to the fascia with 8 to 10 absorbable 3/0 anchoring stitches. Before extubation, an adaptable elastic abdominal wall bandage with Velcro™ was placed on the abdominal wall. All patients wore the adaptable bandage day and night with approximatelly two-hour intervals, twice a day.

### Main post-operative management

Antibiotic prophylaxis was not given for the electively operated patients. After acute surgeries, the patients received empiric amoxicillin-clavulanate 1.2 g three times a day and metronidazole 1 mg/kg/day twice a day intravenously for five days, or amended as per the microbiological culture results^12^^,^ 13. All patients were given 1 × 0.4–1,0 milliliter subcutaneously (depending on body weight) enoxaparine thrombosis prophylaxis for 21 days. For analgesia, non-steroid anti-inflammatory drugs were administered only when needed. Patients were mobilized on the second post-operative day by a physiotherapist.

### Investigated parameters

The average blood loss during surgery was assessed based on the hematocrit (H) values before and 1 hour after surgery (Eq. 1):


$$EBL=EBV\times \ln\;\left(H_{i}/H_{f}\right)$$ (1)

where: EBV: estimated blood loss, mL; EBV: estimated blood volume, mL; H_i,f_: hematocrit initial and final^14^.

On the first, third and fifth post-operative day, intra-abdominal pressure (IAP) was measured by an intra-abdominal pressure monitoring set^15^.

During the first five days, the amount of fluid drained was measured.

On the first, third and fifth post-operative day, patients were asked to evaluate pain intensity on a 10-grade scale by Numerical Rating Scale:

- 1 or 2: none or not significant;
- 3 or 4: mild;
- 5 or 6: moderate;
- 7 or 8: strong;
- 9 or 10: very strong pain.

Superficial and deep surgical infections, seroma/subcutaneous diffuse fluid accumulation were registered.

The patients were asked to assess the pre-operative (seven days before) and the early results of the intervention (30 days after) by a quality-of-life questionnaire as per Ferrans-Powers^16^ quality of life index (QoLi) adapted to this study (nine questions, each scored from 1 to 6, minimun score = 6, maximum score = 54). Computed tomography assays were carried out three months after reconstructions.

Surgical complications as per Clavien-Dindo classification^17^, abdominal wall bulking, and recurrent abdominal wall hernia were registered.

### Follow-up

Follow-up examinations are seen in Table 3.

**Table 3:** Follow-up examinations and its timing during the study.

	Preop.	Post-operative (day)				Post-operative (month)						
		1	3	5		1	3	6	12	24	36	42
Intra-abdominal pressure measure	+	+	+	+		-	-	-	-	-	-	-
Pain numerical rating scale	+	+	+	+		-	+	-	+	-	-	-
Physical examination	+	-	-	-		+	+	+	+	+	+	+
Ultrasonography	-	-	-	-		+	-	+	-	-	-	+
Quality of life index	+	-	-	-		+	-	+	+	+	+	-
Chest Xray	+	-	-	-		-	-	-	+	-	-	-
Computed tomography	+	-	-	-		-	+	-	+	+	+	-

Source: Elaborated by the authors.

## Results

The operation described was performed in all the 22 patients. The causes of acute surgeries were ileum perforation and peritonitis in two cases, and mechanical bowel obstructions in four cases.

The midline abdominal wall defects were reconstructed without tension by releasing the rectus abdominis muscles from lateral direction and turning them over. In all cases, the turned over rectus muscles could be fixed together with interrupted stitches comfortably. Ideally, the reconstructed abdominal wall consisted of three layers:

- Peritoneal surface of the hernia sac;
- Anterior fascia of the rectus muscle;
- The rectus muscles turned over.

**Figure 7:**
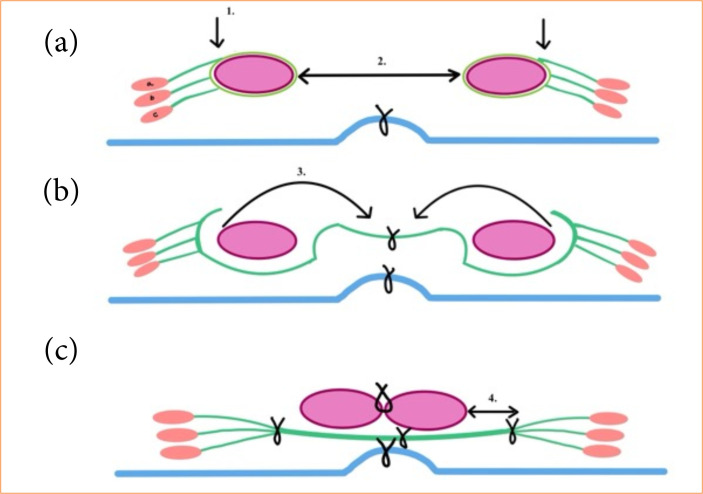
Schematic flow chart of the three crucial steps of the procedure. Cross section view. **(a)** Bilateral incision of the anterior rectus sheath. Reduced peritoneum reconstructed in the midline. **(b)** Midline suture of the turned-over anterior rectus sheath. Turning-over the mobilized rectus muscles. **(c)** Ancoring the lateral edge of the Spigelian belt both sides and midline rectus suture.

Source: Elaborated by the authors.

**Figure 8:**
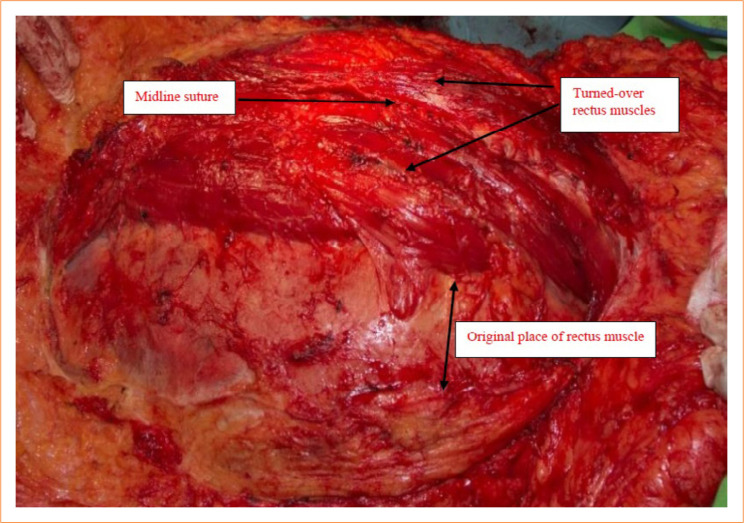
The abdominal wall reconstruction is complete. The bilateral rectus abdominis muscles turned over to the middle are well visible. The muscle is of normal color, its blood supply is adequate. The reconstruction is free from tension in a relaxed condition. The original abdominal wall defect was 547 cm2. The lateral segment (posterior wall of rectus sheath) is intact, with no signs of damage.

Source: Elaborated by the authors.

In Fig. 7, a schematic flow diagram presents the crucial steps of the procedure and the structure of the reconstructed median gap. The muscles remained viable, and their blood supply were satisfactory (Fig. 8). All but one patient tolerated the surgeries excellently. Re-operation was necessary in one case on the first post-operative day due to bleeding, which was a grade IIIb complication as per Clavien-Dindo classification. Mean surgery duration was 127 ± 21 minutes. Mean blood loss during surgery was 351 ± 60.3 mL.

**Figure 9:**
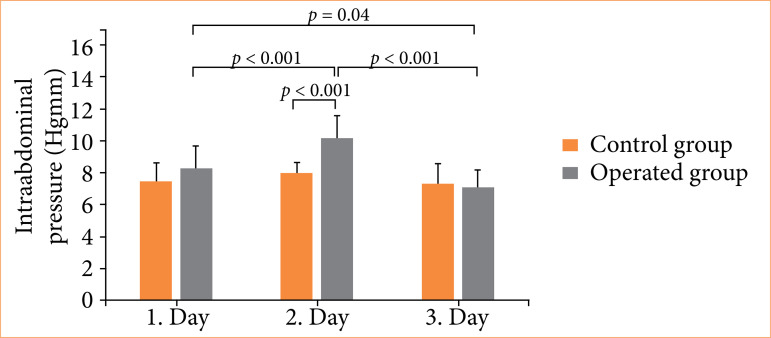
Changes in intra-abdominal pressures on the first, third, and fifth post-operative days compared to those of the control group (20 patients, each operated on superficial femoral artery). The operated and control groups were statistically comparable regarding age, gender, and body mass index. No significant differences were measured between them.

Source: Elaborated by the authors.

Mean post-operative IAP is presented on Fig. 9. While mean intra-abdominal pressure values in the control group did not differ significantly from one another after the surgery, intra-abdominal pressure showed a significant decrease by day 3 in the study population, following a transient elevation after day 1 (day 1 = 8.3 ± 1.4 mmHg vs. day 2 = 10.2 ± 1.4 mmHg; p < 0.001 and day 2 = 10.2 ± 1.4 mmHg vs. day 3 = 7.1 ± 1.1 mmHg; p < 0.001). When the pressure values measured on the first post-operative day were compared to those measured on day 3, we found that there was a significant decrease in intra-abdominal pressure for the study population (day 1 = 8.3 ± 1.4 mmHg vs. day 3 = 7.1 ± 1.1 mmHg; p = 0.04).

Rectus abdominis muscle sheath; Rectus abdominis muscle; 1: place of anterior sheath incision; 2: midline gap; 3: turning over of rectus muscles; 4: Spigelian belt; a: external oblique abdominis muscle; b: internal oblique abdominis muscle; c: transverse abdominis muscle.

A significant IAP elevation was measured for study patients vs. control group only on the third post-operative day (study patients vs. control group = 10.2 ± 1.4 mmHg vs. 8.0 ± 0.7 mmHg, respectively; p < 0.001). The IAP values on days 1 and 5 did not differ significantly. Significant (p < 0.001) decrease of drained fluid was measured between the first and fifth (56 ± 8 vs. 25 ± 7 mL) post-operative day. The last drain was removed on the eigth post-operative day (six to 12 days).

Time until first bowel movement was 3.6 (three to five) days on average. Pain intensity scores were 6.5, 5, and 2.6 on the first, third and fifth post-operative day, respectively. Mean hospitalization time was 6.5 ± 1.24 days. Grade I complications as per Clavien-Dindo classification–superficial wound in infection and diffuse subcutaneous fluid accumulation–developed in two (9.1%) and two cases (9.1%), respectively. The subcutaneous fluid accumulations were treated by percutaneous tapping.

As per the patient satisfaction questionnaire and the quality-of-life test performed four weeks after the surgery, all the 22 patients were satisfied with the results (satisfactory score = 6.0 ± 0.0). The pre-operative QoLi score was 23.3 ± 13.59 seven days before surgery, meanwhile 30 days after the surgery it was 46.7 ± 6.38, p = 0.0013. Mean follow-up time was 25 ± 9 (3–48) months.

Each patient was followed. During follow-up, abdominal wall *bulking* developed in one case was registered in the 13^th^ post-operative month. The patient (53, male) was operated in an elective fashion, his midline gap was 545 cm^2^. *Recurrent* hernia was registered in one case in the 16^th^ post-operative month. The patient (45, male) was operated in an acute fashion, and he was treated on type-2 diabetes mellitus and chronic obstructive pulmonary disease, he was an active smoker. The reconstructed midline gap was 486 cm2. Mean quality-of-life scores in months 6, 12, 18, 24 and 30 were as follows: 47.1 ± 4.2, 45.2 ± 5.3, 48.0 ± 2.9, 47.4 ± 4.5, 46.4 ± 4.8, there was no significant difference between values (p = ns, t-unpaired test). Key findings of the study are summarized in Table 4.

**Table 4:** Key findings of the study.

Postoperative intraabdominal pressure	pressure increase 1^st^, 3^rd^, 5^th^ post-operative day	ns
Postoperative complications (as per Clavien-Dindo)	Grade I (infection/seroma)	4
	Grade II (blood transfusion)	1
	Grade IIIa	0
	Grade IIIb (reoperation, bleeding)	1
	Grade IV	0
	Grade V	0
Bulking formation	13^th^ post-operative month	1
Hernia recurrency	16^th^ post-operative month	1
Quality-of-life index (as per Ferrans-Powers)	significant increase, 30^th^ post-operative day	*p* = 0.0013

Source: Elaborated by the authors.

## Discussion

Several authors devised effective techniques to resolve eventrated abdominal wall hernias and loss of abdominal wall domain^18^^–^ 25. Treatment of such conditions means an explicit challenge for surgeons^26^^,^ 27. Surgical complications and recurrence ratio after the procedures may be as high as 40–50% and 10–30%, respectively^28^. In several cases, implantation of synthetic or biological mesh for reinforcement of the abdominal wall is inevitable. The original component separation technique uses the abdominal wall’s own structures for reconstruction, however the recurrence rate of abdominal wall hernia is relatively high, and the ratio strongly depends on the width of the abdominal gap^29^. Recurrence rates could be reduced to 4% by component separation and implantation of synthetic mesh and to 11% by component separation and implantation of biological mesh^30^.

The trans-abdominal wall traction method is described as a universal solution in acute and elective cases of eventrated abdominal wall hernias^31^. The rate of primary fascial closure can be improved by using botulinum-A toxin after open abdomen treatments^32^. The so-called “open-book” method is applied to the sizeable midline abdominal gap repair, but the method uses only the anterior rectus abdominis fascia without releasing the muscles themselves for reconstruction^33^. Whichever method or combination of methods is applied to cover a large abdominal wall defect, the objective is that the reconstruction should *trigger the least possible increase* in intra-abdominal pressure.

In this study, the midline gap could be covered with the turned-over bilateral rectus muscles without triggering considerable tension in the lateral muscle-aponeurotic elements. Consequently, the intra-abdominal pressure remained in a moderately elevated range. Lateral and partial mobilization of the origin and the insertion of the rectus abdominis muscle and its turning over from lateral towards the midline did not affect the muscle’s blood supply, although muscle innervation was damaged due to the lateral release^34^. The use of botulinum-A toxin and inflation of the abdominal cavity with CO2 was needless.

There was no considerable intra-abdominal pressure elevation after the operations. In our study, grade I and grade IIIb complications were registered.

The most important *advantage* of this method was that it used only autologous tissues for abdominal wall reconstruction. On the downside, this surgery could only be carried out if the bilateral rectus abdominis muscles were of appropriate width. Careful preservation of the rectus muscle blood supply was crucial. The method was not free from lateral segment bulking formation. If the lateralized posterior wall of rectus sheath is lax and/or noticeably damaged, a synthetic mesh implantation for reinforcing the lateral part of abdominal wall (between the semicircular line and the lateral edge of the turned over rectus muscle–this is the so-called Spigelian belt) must be taken into consideration to prevent late post-operative lateral bulking/laxity, perhaps recurrency.

The intra-abdominal pressure remained near in the normal range in the early post-operative period. Only autologous muscle-aponeurotic tissues were used for the reconstruction, without the need for synthetic or biological mesh implantation. In this study, the procedure was not free from complications, however the ratio of adverse events was acceptably low.

According to QoLi scores, the patients were satisfied with the aesthetic and functional results of the procedure. Complete or partial denervation of the rectus abdominis muscle during the procedures did not cause midline weakness and disintegration of the midline, reconstructed abdominal wall. According to the follow-up computer tomography images, a considerable connecting tissue formation could be detected, and this gradual transformation of reconstructed midline structures played a prominent part in maintaining the abdominal wall integrity.

However poorly studied, chronic postsurgical neuropathic pain represents the second most frequent chronic neuropathic pain etiology, probably affecting 0.5–75% of patients with considerable impact on QoLi^35^. There are a variety of options to manage post-operative pain after an eventrated and/or complicated hernia repair, such as epidural catheters, transversus abdominis plane blocks, intravenous narcotics^36^. Tranversus abdominis plane blocks with bupivacaine were inferior compared to epidural anesthesia or intravenous narcotic pain medications in the early postoperative period. Using epidural anesthesia is recommended preventing post-operative pain. Effective pain management causes shorter full mobilization, reduces hospital stay. Patients with *chronic* abdominal wall pain can be treated with transversus abdominis plane or trigger point(s) injections.

Effectivity of the two procedures is different: the ultrasound guided trigger point injections provide significantly effective pain reduction measured by numerical rating scale^37^. Chronic pain was uncharacteristic after this procedure because there was not implanted mesh (possible source of shrinkage and/or infection), and this technique did not cause considerable intra-abdominal pressure elevation (Fig. 9). The neurovascular bundles preservation innervating the medial abdominal wall was an important factor during the procedures since damaging these bundles could be the source of chronic and/or neurogen abdominal post-operative pain.

The established techniques for abdominal wall repair in cases of eventrated and/or complicated hernias are well known (Novitsky^10^, Chevrel^38^^,^ 39, Da Silva^40^, Rives-Stoppa^41^). These original techniques are very important in the armamentarium for reconstruction of loss abdominal wall domain. These procedures have their established place in surgical practice, and using one of them is the question of *meticulous* preoperative *analysis*. The main results and features of the basic operation techniques can be seen in Table 5. These techniques in the treatment of complex midline incisional hernias provide a rationally acceptable low complication rate and a significant improvement in quality of life.

**Table 5:** Comparative analysis of different reconstructive techniques.

Effectiveness	Chevrel safe, very satisfactory results	Da Silva plastic repair of the median/paramedian hernias	Rives-Stoppa excellent long term results	Novitsky minimal major adverse events	Martis for extra-large median gap	Martis in septic cases as well
Complication rate (overall)	22%	5.5–17.3%	12.3–16.1%	4–21٪	18٪	36٪
Bulking formation	0	4%	12–17%	5–17٪	5٪	11٪
Recovery time (weeks)	4 ± 2	4 ± 3	5 ± 2	6 ± 1	9 ± 3	5 ± 2
Recurrency (two year)	3.2%	5–9%	3.2–10%	4.2–15.4%	5–7%	11%
Mesh implantation	yes	no	yes	yes	no	no
Operating time (min)	95 ± 50	125 ± 35	170 ± 15.08	188.8 ± 22.04	127 ± 21	143 ± 44
Covering ability (cm)	13–15	10–12	8.5–14	12.5–14	15–18	10.5–14

Source: Elaborated by the authors.

Three procedures out of six utilize exclusively autologous tissues for reconstruction–Da Silva, bilateral rectus turn-over (Martis), and double layer dermal grafts for complicated and/or large abdominal hernias^42^ (Martis). It is well known the advantages of mesh implantation considering recurrency ratio, however there are situations, when implantation of synthetic mesh(es) could pose a significant source of complications, e.g., infected operating territory, infected mesh implanted in a previous operation, different infected fistulas, skin infections, immune-compromitted patients, incarcerated giant hernias, bowel perforation(s) during adhesiolysis, acute cases etc. In these cases, the reconstructions without implantation of xenografts must be taken into consideration.

Bilateral rectus muscles were able to cover extremely wide midline gap. Increasing of intra-abdominal pressure was insignificant in the postoperative period. However, the procedure was not complication-free, but these complications could be treated within short time and easily. Our technique presented was not free from bulking formation and not recurrence-free, however this ratio was acceptable. Finally, using bilateral rectus muscles for eventrated abdominal hernias corresponds with those considerable endeavours to use xenografts if their advantages are evident.

If the bilateral rectus abdominis muscles are intact and the width of them together is sufficient to cover the midline abdominal wall defect, then turning over the rectus muscles and midline reconstruction can be carried out with technical success^43^.

Primary indication of the procedure presented is the midline abdominal wall loss of domain and eventrated abdominal wall hernia. The procedure can be taken into consideration in acute, emergency surgery as well. If the rectus muscles are inappropriate for covering the gap without tension and/or the quality of the rectus muscles are inadequate, then the method is contraindicated.

## Conclusion

The procedure presented was safe and feasible for reconstruction eventrated and complicated midline abdominal wall hernias in acute and elective cases as well. To apply the method successfully, an abdominal computer tomography examination and assessment of the abdominal wall muscle-aponeurotic elements were decisive. The results–although promising–cannot be generalized yet, and further studies and analysis of the results are necessary.

## Data Availability

All data generated or analyzed during this study are included in this published article. Trial registration: Study unique identifier number (UIN) 1704, http://www.research registry.com
